# Association of intergenerational relationships with cognitive impairment among Chinese adults 80 years of age or older: prospective cohort study

**DOI:** 10.1186/s12877-022-03529-y

**Published:** 2022-11-07

**Authors:** Qingqing Yang, Jinzhu Jia

**Affiliations:** 1 School of Public Health, Peking University, No.38 Xueyuan Road, Beijing, 100871 China; 2 Center for Statistical Science, Peking University, No.5 Summer Palace Road, Beijing, 100871 China

**Keywords:** The oldest-old, Cognitive impairment, Intergenerational relationships, Parental support, Filial support

## Abstract

**Background:**

The oldest-old (aged 80 or older) are the most rapidly growing age group, and they are more likely to suffer from cognitive impairment, leading to severe medical and economic burdens. The influence of intergenerational relationships on cognition among Chinese oldest-old adults is not clear. We aim to examine the association of intergenerational relationships with cognitive impairment among Chinese adults aged 80 or older.

**Methods:**

This was a prospective cohort study, and data were obtained from the Chinese Longitudinal Healthy Longevity Survey, 14,180 participants aged 80 or older with at least one follow-up survey from 1998 to 2018. Cognitive impairment was assessed by the Chinese version of Mini Mental State Examination, and intergenerational relationships were assessed by *getting main financial support from children*, *living with children or often being visited by children,* and *doing housework or childcare*. We used time-varying Cox proportional hazards models to estimate hazard ratios (HRs) and 95% confidence intervals (CIs) of associations between intergenerational relationships and cognitive impairment.

**Results:**

We identified 5443 incident cognitive impairments in the 24-cut-off MMSE cohort and 4778 in the 18-cut-off MMSE cohort between 1998 and 2018. After adjusting for a wide range of confounders, the HR was 2.50 (95% CI: 2.31, 2.72) in the old who received main financial support from children, compared with those who did not. The HR was 0.89 (95% CI: 0.83, 0.95) in the oldest-old who did housework or childcare, compared with those who did not. However, there were no significant associations between older adults’ cognitive impairments and whether they were living with or often visited by their children. Our findings were consistent in two different MMSE cut-off values (24 vs. 18) for cognitive impairment.

**Conclusions:**

Sharing housework or childcare for children showed a protective effect on older adults’ cognitive function, whereas having children provide primary financial support could increase the risk for cognitive impairments. Our findings suggest that governments and children should pay more attention to older adults whose main financial sources from their children. Children can arrange some easy tasks for adults 80 years of age or older to prevent cognitive impairments.

**Supplementary Information:**

The online version contains supplementary material available at 10.1186/s12877-022-03529-y.

## Introduction

Population aging has become one of the severest challenges worldwide, including in China. Frail health has a bad influence on quality of life among the oldest-old (aged 80 or older). Cognitive impairment is often regarded as one of the most serious factors of health, because it can cause disability in learning, language, memory, etc. It is meaningful to reduce the incidence of cognitive impairment, which can lighten the burden of social care systems. Families have played a basic role in elderly health, so intergenerational relationships—an important branch of family relationships—have become a hot topic in the healthy study of older adults. Most existing literature provides support for the association between intergenerational support and morbidity or mortality, but few studies have examined cognitive function [1–4].

Some studies reported that the elderly who gave their children support were more likely to be cognitively impaired, because informal caregivers experienced more stress than non-caregivers [5–7]. Similarly, Minkler and Fuller-Thomson, etc., concluded that grandparents who were the primary caregivers for their grandchildren were more likely to have depression and a higher risk of cognitive impairment than grandparents who did not undertake primary caregiving responsibilities [8]. However, some longitudinal-sectional studies found that informal caregiving, in terms of looking after someone, could be beneficial for cognitive function, at least for female caregivers [9, 10]. Individuals who lived alone had higher risks of dementia than those who lived with others (e.g., spouse, children, caretaker); older adults who kept in contact with their family also had better mental health [11, 12]. However, in some Western developed countries, the old who lived alone thought they had more free time to enjoy life, so they had good health conditions [13]. Positive or negative relationship qualities in two or three generations of family members may also influenced older adults’ psychological well-being [14–17].

In China, studies on intergenerational support have mostly focused on some special elderly groups, such as empty nesters, the old in rural areas or some provinces and so on [18–21]. Both longitudinal and cross-sectional studies found it beneficial for cognitive function of the oldest-old in Sichuan, Anhui provinces and central China who obtained financial support from their children [18, 22–24]; however, some found that receiving instrumental support from children could enhance the speed of developing cognitive impairment in elderly individuals [18]. Although elderly people living with children were more likely to receive support, intergenerational conflicts and lack of privacy may lower their psychological health [21, 25]. Some studies suggested that marital status might change this relation; for example, coresidence with adult children was beneficial for the widowed compared with the married old [26].

We attempted to use CLHLS data to explore the association of intergenerational relationships with cognitive function overcoming the previous limitations of small sample sizes, special groups, cross-sectional designs and so on [20–23, 26].

## Methods

### Study participants

This study analyzed data obtained from the Chinese Longitudinal Healthy Longevity Surveys (CLHLS) between 1998 and 2018. The CLHLS is a nationwide survey covering 23 out of 31 provinces in China, and has the largest samples of the oldest-old (aged 80 or older). The first wave of CLHLS was in 1998 containing 9093 participants, and had follow-up surveys or recruits in 2000, 2002, 2005, 2008, 2011, 2014 and 2018, covering approximately 85% of the total population. Information was obtained through face-to-face questionnaire interviews conducted by trained staff from the county Centers for Disease Control and Prevention, and a physical health examination was administered by a trained investigator. More details of the CLHLS can be seen in previous studies [27, 28]. The Research Ethics Committees of Peking University and Duke University granted approval for the protection of Human Subjects for the Chinese Longitudinal Healthy Longevity Surveys, including the collection of the data used for the present study. The survey respondents signed written consent forms before participation. This dataset can be accessed via Peking University Open Research Data Platform.

**Fig. 1:**
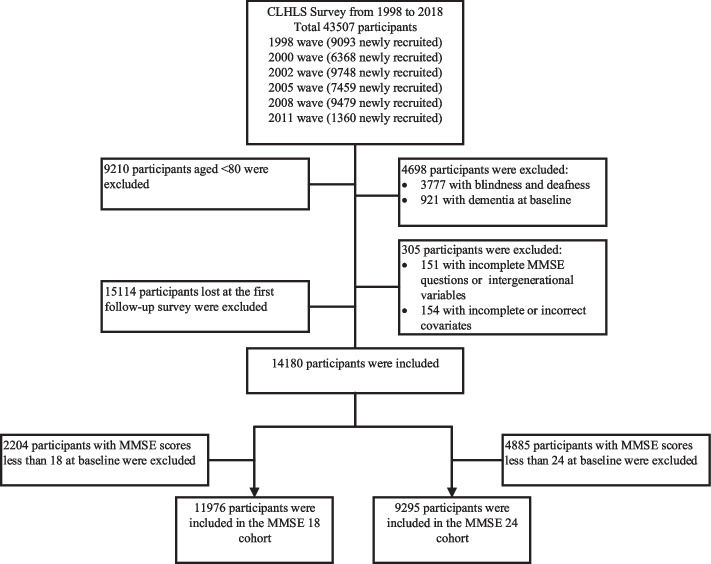
Flow chart of the study population in CLHLS

In our study, we selected a total of 43,507 participants newly recruited in 1998, 2000, 2002, 2005, 2008 and 2011, and the latest follow-up year was 2018. Of these, we excluded: a) 9210 participants aged < 80 years; b) 4698 with blindness, deafness and dementia at baseline; c) 305 with incomplete information in key variables; and d) 15,114 lost at the first follow-up survey. According to the different cut-off values for the Chinese version of Mini Mental State Examination (MMSE) scores for cognitive impairment, we further excluded 4885 and 2204 participants with cognitive impairment at baseline in the cut-off of 24 and 18 cohorts, respectively. Finally, there were 11,976 in the MMSE 18 cohort and 9295 in the MMSE 24 cohort (Fig. 1).

### Measurement of cognitive impairment

Cognitive function was measured by the Chinese version of MMSE based on MMSE of Folstein and other researchers [29], which has proven to be reliable and valid [30]. The Chinese MMSE had 24 questions covering orientation, memory, attention, calculation and language. Except for the item ‘tell names of food in one minute’, which was scored from 0 to 7, each item was 0 or 1, where 0 indicated the disability to perform the task, and otherwise 1. Total scores ranged from 0 to 30, with higher scores indicating better cognitive function [31]. According to Folstein et.al, cognitive functioning could be classified into 4 levels: cognition severely impaired (score 0–9), moderately impaired (score 10–17), slightly impaired (score 18–23), and unimpaired (score 24–30). Some studies also chose 18 as the cut-off value for those with little education [32–34]. In our study, we chose 24 as the cut-off point to show our main findings, and the results of the 18 cut-off point can be seen in Supplementary. Our results were similar in the two cut-off values.

### Measurement of intergenerational relationships

Intergenerational relationships included parental support and filial support. We used two indexes –*main financial support* and *living or being visited* – to define filial support. In the questionnaire, *main financial support* came from the question that main source of financial support. If the elderly got main financial support from their children or grandchildren, *main financial support* was yes; otherwise it was no. If the elderly lived with their children, grandchildren, great grandchildren and spouses of these descendants mentioned above or were often visited by children, *living or being visited* was yes; otherwise it was no.

*Housework or childcare* was used to reflect parental support, and if the elderly lived with their descendants and their answer for—do you do housework or childcare at present- was at least once a week or more, this was yes; otherwise it was no.

### Covariates

Covariates considered were defined as follows: sociodemographic information including age (continuous variable), gender (female or male), residence(urban or rural), education (literacy, receiving any formal education > 1 year; illiteracy, receiving formal education < 1 year), and marital status (married or not); lifestyle behaviors including smoking status (current smoker, former smoker, or nonsmoker), alcohol status (current drinker, former drinker, or nondrinker), and regular exercise (no or yes); prevalence of hypertension(no or yes), diabetes(no or yes), heart disease (no or yes), cerebrovascular disease (no or yes), respiratory disease (no or yes) and cancer(no or yes), and comorbidity (yes, with at least one disease above; no, without any disease above).

Disability in activities of daily living (ADL) was defined as the inability to perform any task (eating, toileting, bathing, dressing, indoor activities, and continence) independently.

Body mass index (BMI) was a continuous variable, calculated as weight in kilograms divided by height in meters squared. If direct height was not acquired, height was estimated by the knee height (vertical distance from the sole of the foot to the upper surface of the knee with the knee and ankle each fixed to a 90° angle) using equations (men, height = 67.78 + 2.01 × knee height; women, height = 74.08 + 1.81 × knee height).

For missing data, we performed multiple imputation methods.

### Statistical analysis

Categorical variables were described by frequency (percentages), and continuous variables were described by mean (standard deviation (SD)). χ2 or Fisher’s exact test was used to compare the proportions in different subgroups; t tests were used for continuous variables.

Cox proportional hazards regression with time-varying covariates was used to estimate the association between intergenerational relationships and cognitive impairment [35]. Follow-up time was used as the timescale, which was calculated as the time from recruitment to the first occurrence of cognitive impairment or death or the last follow-up survey.

We first examined the association between intergenerational relationships and cognitive impairment using the crude hazard ratio (HR). Then we further adjusted for age, gender, residence, marital status and education. Finally, models were further adjusted for smoking, drinking status, exercise, ADL disability, BMI, comorbidity and baseline Chinese MMSE scores. Note that the intergenerational relationship variables, ADL, age and comorbidity were time varying in the models.

To examine whether the association changed in different groups, we performed the analyses in different subgroups. We also compared our results with traditional Cox regression models to show the influence of varying intergenerational relationships. Finally, we furtherly reselected 18 as the cut-off value for cognitive impairment to examine whether our findings were sensitive to cut-off points of MMSE.

We used R version 4.1.1 for statistical analyses, and the results were statistically significant at *P* < 0.05 for all analyses.

## Results

### Baseline characteristics of participants

We identified 5443 incident cognitive impairment in the 24-cut-off value cohort and 4778 in the 18-cut-off value cohort between 1998 and 2018. In the 24-cut-off value cohort, 2663 and 1189 participants died and were lost in the follow-up surveys, respectively; 4974 died and 2224 were lost in the follow-up surveys in the 18-cut-off value cohort. The mean of follow-up time was 5.45 (SD: 4.95) and 6.15 (SD: 5.34) years in the 24-cut-off and 18-cut-off cohorts, respectively. The oldest-old adults who developed cognitive impairment tended to be older, illiterate and female, live in rural areas, and have no regular exercise and lower BMI (Table 1 & Table S1).

**Table 1:** Baseline characteristics of the cohort with cut-off value 24 for MMSE scores, 1998–2018

		Cognitive impairment		
	Overall	No	Yes	*P* value
	*N* = 9295	*N* = 3852	*N* = 5443	
Age (mean (SD))	88.35 (6.66)	86.96 (6.13)	89.34 (6.85)	< 0.001
Age group (%)				< 0.001
80–90	5569 (59.91)	2632 (68.33)	2937 (53.96)	
90–100	2749 (29.58)	975 (25.31)	1774 (32.59)	
100 +	977 (10.51)	245 ( 6.36)	732 (13.45)	
Follow-up time (mean (SD))	5.45 (4.95)	7.16 (6.10)	4.23 (3.46)	< 0.001
Education (%)				< 0.001
Illiteracy	5760 (61.97)	1992 (51.71)	3768 (69.23)	
Literacy	3535 (38.03)	1860 (48.29)	1675 (30.77)	
Gender (%)				< 0.001
Female	4663 (50.17)	1477 (38.34)	3186 (58.53)	
Male	4632 (49.83)	2375 (61.66)	2257 (41.47)	
Marital status (%)				0.193
No	106 ( 1.14)	51 ( 1.32)	55 ( 1.01)	
Yes	9189 (98.86)	3801 (98.68)	5388 (98.99)	
Residence (%)				< 0.001
Urban	4266 (45.90)	1865 (48.42)	2401 (44.11)	
Rural	5029 (54.10)	1987 (51.58)	3042 (55.89)	
Smoke (%)				< 0.001
No	5778 (62.16)	2127 (55.22)	3651 (67.08)	
Current	2155 (23.18)	1020 (26.48)	1135 (20.85)	
Former	1362 (14.65)	705 (18.30)	657 (12.07)	
Drink (%)				< 0.001
No	6171 (66.39)	2406 (62.46)	3765 (69.17)	
Current	2156 (23.20)	1002 (26.01)	1154 (21.20)	
Former	968 (10.41)	444 (11.53)	524 ( 9.63)	
Exercise (%)				< 0.001
No	5828 (62.70)	2216 (57.53)	3612 (66.36)	
Yes	3467 (37.30)	1636 (42.47)	1831 (33.64)	
ADL disability (%)				< 0.001
No	8026 (86.35)	3446 (89.46)	4580 (84.14)	
Yes	1269 (13.65)	406 (10.54)	863 (15.86)	
BMI (mean (SD))	19.99 (4.24)	20.22 (4.24)	19.82 (4.23)	< 0.001
Hypertension (%)				0.01
No	7805 (83.97)	3189 (82.79)	4616 (84.81)	
Yes	1490 (16.03)	663 (17.21)	827 (15.19)	
Diabetes (%)				0.223
No	9179 (98.75)	3797 (98.57)	5382 (98.88)	
Yes	116 ( 1.25)	55 ( 1.43)	61 ( 1.12)	
Heart disease (%)				0.036
No	8604 (92.57)	3539 (91.87)	5065 (93.06)	
Yes	691 ( 7.43)	313 ( 8.13)	378 ( 6.94)	
Cerebrovascular disease (%)				0.621
No	9004 (96.87)	3736 (96.99)	5268 (96.78)	
Yes	291 ( 3.13)	116 ( 3.01)	175 ( 3.22)	
Respiratory disease (%)				0.016
No	8235 (88.60)	3376 (87.64)	4859 (89.27)	
Yes	1060 (11.40)	476 (12.36)	584 (10.73)	
Cancer (%)				0.818
No	9271 (99.74)	3841 (99.71)	5430 (99.76)	
Yes	24 ( 0.26)	11 ( 0.29)	13 ( 0.24)	
Comorbidity (%)				< 0.001
No	6397 (68.82)	2552 (66.25)	3845 (70.64)	
Yes	2898 (31.18)	1300 (33.75)	1598 (29.36)	
Housework or childcare (%)				> 0.999
No	7078 (76.15)	2933 (76.14)	4145 (76.15)	
Yes	2217 (23.85)	919 (23.86)	1298 (23.85)	
Main financial support (%)				< 0.001
No	3248 (34.94)	1652 (42.89)	1596 (29.32)	
Yes	6047 (65.06)	2200 (57.11)	3847 (70.68)	
Living or being visited (%)				0.31
No	704 (7.57)	305 (7.92)	399 (7.33)	
Yes	8591 (92.43)	3547 (92.08)	5044 (92.67)	
MMSE scores (mean (SD))	27.64 (1.88)	27.99 (1.78)	27.40 (1.91)	< 0.001

The older adults—living with children or often being visited by children, getting main financial support from children, and sharing housework or childcare—tend to be illiterate, female, in rural areas, and do not have regular exercise, ADL disability and comorbidities. Those who shared housework or childcare were younger, which was different from those receiving main financial support from children and living with or being visited by children (Table S2-S7).

### Associations between intergenerational relationships and cognitive impairment

Main financial support from descendants was associated with a higher risk of incident of cognitive impairment among the oldest-old adults. After adjusting for a wide range of confounders, compared with those who got main support from others, the HR was 2.50 (95% CI: 2.31, 2.72) in the elderly who received main financial support from children. There was no significant correlation between older adults’ cognitive impairments and whether they were living with or visited by their children. However, sharing housework or childcare can decrease the risk for cognitive impairment by 11% (HR 0.89, 95% CI: 0.83, 0.95), compared with those who did not. The results were similar in the 18-cut-off cohort (Table 2 & Table S8).

**Table 2:** Association between intergenerational relationships and cognitive impairment with cut-off value 24 for MMSE scores

	Model 1		Model 2		Model 3	
	Crude HR (95% CI)	*P* value	HR (95% CI)	*P* value	HR (95% CI)	*P* value
Main financial support		< 0.001		< 0.001		< 0.001
No	1 Reference		1 Reference		1 Reference	
Yes	3.00 (2.77, 3.24)		2.55 (2.35, 2.77)		2.50 (2.31, 2.72)	
Living or being visited		0.278		0.807		0.793
No	1 Reference		1 Reference		1 Reference	
Yes	1.06 (0.96, 1.17)		1.01 (0.91, 1.13)		1.01 (0.91, 1.13)	
Housework or childcare		0.001		< 0.001		< 0.001
No	1 Reference		1 Reference		1 Reference	
Yes	0.90 (0.84, 0.96)		0.86 (0.81, 0.92)		0.89 (0.83, 0.95)	

*Abbreviations*: *HR* Hazard ratio, *CI* Confidence interval

Model 2 were adjusted for age, gender, residence, marital status and education

Model 3 were further adjusted for smoking, drinking status, exercise, ADL disability, BMI, comorbidity and baseline Chinese MMSE scores

### Sensitivity analyses

**Fig. 2:**
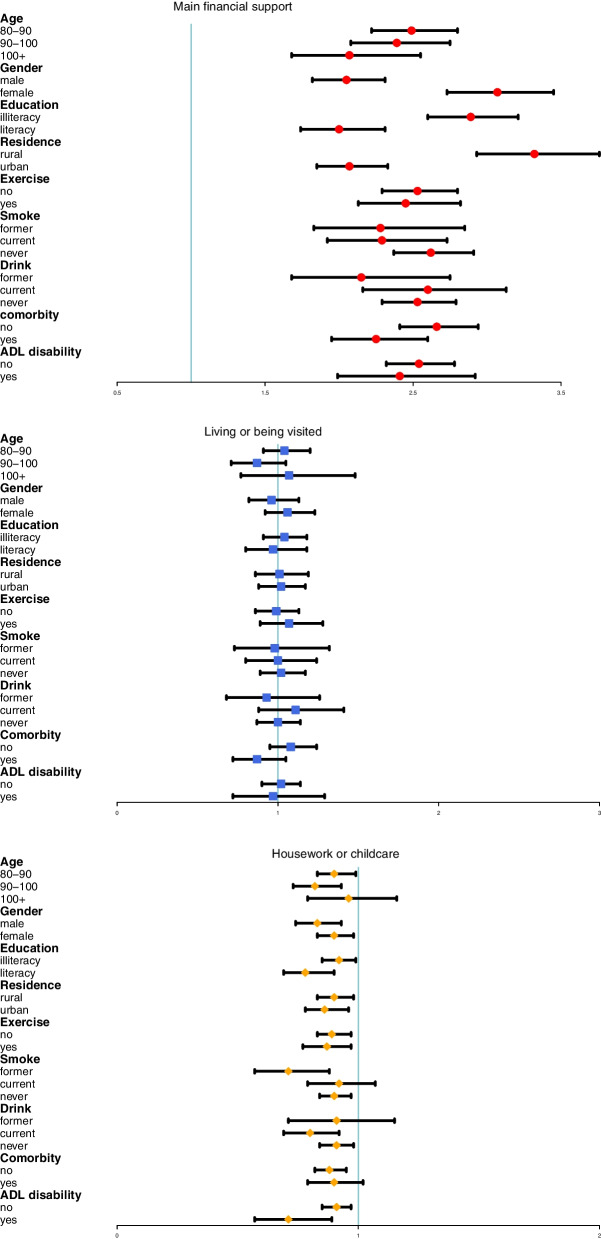
Association between intergenerational relationships and cognitive impairment in different subgroups

We conducted stratified analyses to find potential susceptible subpopulations. For *main financial support*, the risk was higher in the younger age group, illiterate, female and rural areas. For *sharing housework or childcare*, the protective effect was stronger in the male, literate, former smoker and those with ADL disability (Fig. 2).

We also compared the results of the time-varying Cox regression model with traditional Cox regression. The cumulative cognitive impairment curves were shown in Fig. S1. Without considering changes of intergenerational relationships, the HR was 1.26 (95% CI: 1.18, 1.34) and 0.92 (95% CI: 0.86, 0.98) in the main financial support from children group and the housework or childcare group, respectively. The results of *living or being visited* were not significant with cognitive impairment, which was also consistent with time-varying Cox proportional hazards models (Table 3 & Table S9).

**Table 3:** Association between intergenerational relationships and cognitive impairment with cut-off value 24 for MMSE scores using traditional Cox regression

	Model1		Model2		Model3	
	Crude HR (95% CI)	*P* value	HR (95% CI)	*P* value	HR (95% CI)	*P* value
Main financial support		< 0.001		< 0.001		< 0.001
No	1 Reference		1 Reference		1 Reference	
Yes	1.64 (1.55, 1.74)		1.28 (1.20, 1.37)		1.26 (1.18, 1.34)	
Living or being visited		0.625		0.674		0.793
No	1 Reference		1 Reference		1 Reference	
Yes	1.03 (0.93, 1.14)		0.98 (0.88, 1.09)		0.97 (0.87, 1.08)	0.526
Housework or childcare		0.002		< 0.001		0.009
No	1 Reference		1 Reference		1 Reference	
Yes	0.91 (0.85, 0.96)		0.88 (0.83, 0.94)		0.92 (0.86, 0.98)	

*Abbreviations*: *HR* Hazard ratio, *CI* Confidence interval

Model 2 were adjusted for age, gender, residence, marital status and education

Model 3 were further adjusted for smoking, drinking status, exercise, ADL disability, BMI, comorbidity and baseline Chinese MMSE scores

## Discussion

### Principal findings

In this longitudinal study of > 9000 Chinese adults 80 years of age or older followed for a mean of 5 years more, the main source of financial support from descendants was a risk factor for cognitive impairment among the elderly, but sharing housework or childcare for descendants can benefit cognitive function. Living with or often being visited by descendants in our study was not significantly associated with cognitive impairment. Our findings were consistent in two different MMSE cut-off values (24 vs. 18) for cognitive impairment.

### Comparison with previous studies

For financial support, many studies show that there is a positive association between intergenerational financial exchange and cognitive impairment in China and Western countries [36–40]. Some studies also showed that receipt of monetary support without being able to give financial support may be bad for cognitive function [18, 41, 42]. The reason why the association in our study was negative may be that we did not consider financial support from the old to their children and only considered the highest level of financial support resource. The elderly receiving the most money from their children may have little pension, insurance and other welfare. Even if they have welfare, severe diseases or other reasons can also cause main financial support from children. Under the above conditions and Chinese traditional parents’ role, the elderly who could not give adequate help to their children will feel stressed and have terrible mental health. The negative effect was higher in rural areas and illiterate people; one explanation for this is that these kinds of oldest-old adults usually did not have any pension or medical insurance, which means that they were almost wholly dependent on support from children with little support to children [18]. Females also have a higher risk. Because they usually raised children without working outside, they did have any financial resources to support themselves when they were old [43]. When they were older, they did have the ability to help their children, which caused more stress.

For living with children or often being visited by children, their influence on cognitive function are different. In Western developed countries, most studies show that cognitive function in the old who live alone is better [44], and they are also more likely to find an earlier stage of dementia [11]. Due to economic and cultural status, the elderly in Western countries tend to live alone. They pay more attention to their health status than their families. In Asia, researches in some countries show that elderly individuals living with children are associated with a higher risk of depression [45–47]. However, in China, the results were different. Some studies think the oldest old living with their children tend to have better psychological well-being [26, 48], because they will have more opportunities to gain more physical and emotional support from their children. But some researchers did not find that older parents who lived in three-generation households can obtain benefits [49]. We did not find a significant effect of living or being visited, and the reasons may be as follows. First, coresidence with children is the most prevalent living arrangement for the Chinese oldest-old [26, 48, 50], but with the rapid economic and educational development, independent living arrangement increases, which may suggest that living arrangements of the Chinese oldest-old are partially getting Westernized [50] and the effect in this situation is not significant. Second, if the old are in poor health status, they tend to live with children or often be visited by children, which may offset their benefits. Third, intergenerational relationship qualities across different generations influence elderly adults’ psychological condition. There may be both negative and positive relationship qualities, which can influence the effect of living with or being visited by descendants [14, 15]. Fourth, we do not consider the role of their spouses, which may make the results different.

Previous studies on caring for grandchildren have varied, with both positive and negative influences on older adults. In the United States, most studies show that extensive care for a grandchild is associated with negative health outcomes [8, 51–53]. They think grandchild care can cause changes in lifestyle, relationships, and social roles, and make them feel stressed. But there also studies show that there were lower or no risk of health problems [54, 55]. In contrast to most negative results in the Western countries, psychological benefits were shown in most Asian countries, especially in China [49, 56–61]. Our results were consistent with previous findings in China. The benefits of doing housework or childcare among Chinese oldest-old adults may be explained by the following reasons. Physical and cognitive disabilities increase with aging, which means that oldest-old adults will need more support from their children, but give their children less support. Traditional Chinese parents usually regard giving supports to children as an obligation, and they will feel guilty and worthless, especially oldest-old adults in rural areas who usually do not have enough pensions. Therefore, oldest-old adults will feel a more meaningful sense of social participation, self-confidence, self-value and self-acceptance, if they can still do housework or childcare for their offspring. At the same time, older adults usually share some wealth of skills, experiences and valuable advice in this process. Then they experience appreciation and gratitude from younger generations for their wisdom, sense of importance and value, which will enhance their positive emotions and satisfaction [62–64]. In addition, doing housework or childcare were cognitively complex tasks that needed attention, alertness and consideration. The more older adults do, the better their cognition. In our results, both men and women can benefit from housework or childcare; in particular, the benefits were greater among males. In traditional Chinese families, women usually do more housework and raise their children, while men generally earn money outside [65]. Therefore, men doing more tasks in the family may have a sense of fulfillment, which means they can benefit more from housework or childcare than women. The results in rural areas were also consistent with previous studies [43, 59].

### Strengths and limitations of this study

Our study has some notable strengths. First, our study had a prospective study design, a large sample size covering most provinces in China, and a longer follow-up time. Second, we used Cox proportional hazards regression with time-varying covariates. Cox models only considering the status of intergenerational relationships at baseline can underestimate the protective effect of housework or childcare and the risk of main financial support. Third, we obtained robust results in different cut-off values for MMSE scores to define cognitive impairment.

On the other hand, our study has several limitations. First, CLHLS does not include all provinces, so it is not entirely representative of China. Second, although we adjusted for a wide range of potential confounders, we cannot exclude the possibility of residual confounding.

## Conclusions

In this longitudinal prospective study among Chinese oldest-old adults, sharing housework or childcare for children showed a protective effect on older adults’ cognitive function, and getting the main source of financial support from children could increase the risk for cognitive impairments. Our findings were consistent in two cohorts with different MMSE cut-off values for cognitive impairments. The findings suggest that governments and children should pay more attention to older adults whose main financial sources from children. Children can arrange some easy tasks for adults 80 years of age or older to prevent cognitive impairments.

## Supplementary Information

**Additional file 1: Table S1.** Baseline characteristics of the cohort with cut-off value 18 for MMSE scores, 1998-2018. **Table S2.** Baseline characteristics of the cohort with cut-off value 24 for MMSE scores from housework or childcare, 1998-2018. **Table S3.** Baseline characteristics of the cohort with cut-off value 24 for MMSE scores from main financial support, 1998-2018. **Table S4.** Baseline characteristics of the cohort with cut-off value 24 for MMSE scores from living or being visited, 1998-2018. **Table S5.** Baseline characteristics of the cohort with cut-off value 18 for MMSE scores from housework or childcare, 1998-2018. **Table S6.** Baseline characteristics of the cohort with cut-off value 18 for MMSE scores from main financial support, 1998-2018. **Table S7.** Baseline characteristics of the cohort with cut-off value 18 for MMSE scores from living or being visited, 1998-2018. **Table S8.** Association between intergenerational relationships and cognitive impairment with cut-off value 18 for MMSE scores. **Table S9.** Association between intergenerational relationships and cognitive impairment with cut-off value 18 for MMSE scores using traditional Cox regression. **Figure S1.** Cumulative cognitive impairment curves in intergenerational relationships variables.

## Data Availability

All the data involved in this article can be obtained online (https://opendata.pku.edu.cn/dataset.xhtml?persistentId=doi:10.18170/DVN/WBO7LK).

## Abbreviations

CLHLS: The Chinese Longitudinal Healthy Longevity Survey
MMSE: Mini Mental State Examination
HR: Hazard ratio
CI: Confidence interval
ADL: Disability in activities of daily living
BMI: Body mass index
SD: Standard deviation
