# Supplementary material for: Association of intergenerational relationships with cognitive impairment among Chinese adults 80 years of age or older: prospective cohort study
Source: BMC Geriatr. 2022 Nov 7;22:838. doi: 10.1186/s12877-022-03529-y (PMC9639315; doi:10.1186/s12877-022-03529-y)
Supplement: Supplementary file 1 — Additional file 1: Table S1. Baseline characteristics of the cohort with cut-off value 18 for MMSE scores, 1998-2018. Table S2. Baseline characteristics of the cohort with cut-off value 24 for MMSE scores from housework or childcare, 1998-2018. Table S3. Baseline characteristics of the cohort with cut-off value 24 for MMSE scores from main financial support, 1998-2018. Table S4. Baseline characteristics of the cohort with cut-off value 24 for MMSE scores from living or being visited, 1998-2018. Table S5. Baseline characteristics of the cohort with cut-off value 18 for MMSE scores from housework or childcare, 1998-2018. Table S6. Baseline characteristics of the cohort with cut-off value 18 for MMSE scores from main financial support, 1998-2018. Table S7. Baseline characteristics of the cohort with cut-off value 18 for MMSE scores from living or being visited, 1998-2018. Table S8. Association between intergenerational relationships and cognitive impairment with cut-off value 18 for MMSE scores. Table S9. Association between intergenerational relationships and cognitive impairment with cut-off value 18 for MMSE scores using traditional Cox regression. Figure S1. Cumulative cognitive impairment curves in intergenerational relationships variables. [file 12877_2022_3529_MOESM1_ESM.docx]

**Supplementary**

**Association of Intergenerational Relationships with Cognitive Impairment among** **Chinese Adults 80 Years of Age or Older: Prospective Cohort Study**

**Table of contents**

**Table S1.** Baseline characteristics of the cohort with cut-off value 18 for MMSE scores, 1998-2018.

**Table S2.** Baseline characteristics of the cohort with cut-off value 24 for MMSE scores from housework or childcare, 1998-2018.

**Table S3.** Baseline characteristics of the cohort with cut-off value 24 for MMSE scores from main financial support, 1998-2018.

**Table S4.** Baseline characteristics of the cohort with cut-off value 24 for MMSE scores from living or being visited, 1998-2018.

**Table S5.** Baseline characteristics of the cohort with cut-off value 18 for MMSE scores from housework or childcare, 1998-2018.

**Table S6.** Baseline characteristics of the cohort with cut-off value 18 for MMSE scores from main financial support, 1998-2018.

**Table S7.** Baseline characteristics of the cohort with cut-off value 18 for MMSE scores from living or being visited, 1998-2018.

**Table S8.** Association between intergenerational relationships and cognitive impairment with cut-off value 18 for MMSE scores.

**Table S9.** Association between intergenerational relationships and cognitive impairment with cut-off value 18 for MMSE scores using traditional Cox regression.

**Figure S1.** Cumulative cognitive impairment curves in intergenerational relationships variables.

**Table S1. Baseline characteristics of the cohort with cut-off value 18 for MMSE scores, 1998-2018.**

|  |  | Cognitive impairment | |  |
| --- | --- | --- | --- | --- |
|  | Overall | No | Yes | P value |
|  | N=11976 | N=7198 | N=4778 |  |
| Age (mean (SD)) | 89.08 (6.91) | 87.86 (6.48) | 90.93 (7.13) | <0.001 |
| Age group (%) |  |  |  | <0.001 |
| 80-90 | 6659 (55.60) | 4521 (62.81) | 2138 (44.75) |  |
| 90-100 | 3719 (31.05) | 2036 (28.29) | 1683 (35.22) |  |
| 100+ | 1598 (13.34) | 641 ( 8.91) | 957 (20.03) |  |
| Follow-up time (mean (SD)) | 6.15 (5.34) | 7.35 (6.10) | 4.34 (3.15) | <0.001 |
| education (%) |  |  |  | <0.001 |
| illiteracy | 7992 (66.73) | 4401 (61.14) | 3591 (75.16) |  |
| literacy | 3984 (33.27) | 2797 (38.86) | 1187 (24.84) |  |
| Gender (%) |  |  |  | <0.001 |
| Female | 6554 (54.73) | 3419 (47.50) | 3135 (65.61) |  |
| Male | 5422 (45.27) | 3779 (52.50) | 1643 (34.39) |  |
| Marital status (%) |  |  |  | 0.075 |
| No | 137 ( 1.14) | 93 ( 1.29) | 44 ( 0.92) |  |
| Yes | 11839 (98.86) | 7105 (98.71) | 4734 (99.08) |  |
| Residence (%) |  |  |  | <0.001 |
| urban | 5285 (44.13) | 3318 (46.10) | 1967 (41.17) |  |
| rural | 6691 (55.87) | 3880 (53.90) | 2811 (58.83) |  |
| Smoke (%) |  |  |  | <0.001 |
| no | 7722 (64.48) | 4357 (60.53) | 3365 (70.43) |  |
| current | 2632 (21.98) | 1729 (24.02) | 903 (18.90) |  |
| former | 1622 (13.54) | 1112 (15.45) | 510 (10.67) |  |
| Drink (%) |  |  |  | <0.001 |
| no | 8067 (67.36) | 4689 (65.14) | 3378 (70.70) |  |
| current | 2694 (22.49) | 1740 (24.17) | 954 (19.97) |  |
| former | 1215 (10.15) | 769 (10.68) | 446 ( 9.33) |  |
| Exercise (%) |  |  |  | <0.001 |
| No | 7854 (65.58) | 4461 (61.98) | 3393 (71.01) |  |
| Yes | 4122 (34.42) | 2737 (38.02) | 1385 (28.99) |  |
| ADL disability (%) |  |  |  | <0.001 |
| No | 10004 (83.53) | 6253 (86.87) | 3751 (78.51) |  |
| Yes | 1972 (16.47) | 945 (13.13) | 1027 (21.49) |  |
| BMI (mean (SD)) | 19.76 (4.21) | 19.93 (4.22) | 19.50 (4.19) | <0.001 |
| Hypertension (%) |  |  |  | <0.001 |
| No | 10054 (83.95) | 5961 (82.81) | 4093 (85.66) |  |
| Yes | 1922 (16.05) | 1237 (17.19) | 685 (14.34) |  |
| Diabetes (%) |  |  |  | 0.104 |
| No | 11836 (98.83) | 7104 (98.69) | 4732 (99.04) |  |
| Yes | 140 ( 1.17) | 94 ( 1.31) | 46 ( 0.96) |  |
| Heart disease (%) |  |  |  | 0.175 |
| No | 11113 (92.79) | 6660 (92.53) | 4453 (93.20) |  |
| Yes | 863 ( 7.21) | 538 ( 7.47) | 325 ( 6.80) |  |
| Cerebrovascular disease (%) |  |  |  | 0.281 |
| No | 11599 (96.85) | 6982 (97.00) | 4617 (96.63) |  |
| Yes | 377 ( 3.15) | 216 ( 3.00) | 161 ( 3.37) |  |
| Respiratory disease (%) |  |  |  | 0.009 |
| No | 10647 (88.90) | 6355 (88.29) | 4292 (89.83) |  |
| Yes | 1329 (11.10) | 843 (11.71) | 486 (10.17) |  |
| Cancer (%) |  |  |  | 0.685 |
| No | 11947 (99.76) | 7179 (99.74) | 4768 (99.79) |  |
| Yes | 29 ( 0.24) | 19 ( 0.26) | 10 ( 0.21) |  |
| Comorbidity (%) |  |  |  | <0.001 |
| No | 8272 (69.07) | 4833 (67.14) | 3439 (71.98) |  |
| Yes | 3704 (30.93) | 2365 (32.86) | 1339 (28.02) |  |
| Housework or childcare (%) |  |  |  | 0.136 |
| No | 9287 (77.55) | 5548 (77.08) | 3739 (78.25) |  |
| Yes | 2689 (22.45) | 1650 (22.92) | 1039 (21.75) |  |
| Main financial support (%) |  |  |  | <0.001 |
| No | 3804 (31.76) | 2603 (36.16) | 1201 (25.14) |  |
| Yes | 8172 (68.24) | 4595 (63.84) | 3577 (74.86) |  |
| Living or being visited (%) |  |  |  | 0.219 |
| No | 920 (7.68) | 571 (7.93) | 349 (7.30) |  |
| Yes | 11056 (92.32) | 6627 (92.07) | 4429 (92.70) |  |
| MMSE scores (mean (SD)) | 26.13 (3.36) | 26.69 (3.11) | 25.29 (3.54) | <0.001 |

**Table S2. Baseline characteristics of the cohort with cut-off value 24 for MMSE scores from housework or childcare, 1998-2018.**

|  |  | housework or childcare | |  |
| --- | --- | --- | --- | --- |
|  | Overall | No | Yes | p |
|  | N=9295 | N=7078 | N=2217 |  |
| Age (mean (SD)) | 88.35 (6.66) | 88.64 (6.75) | 87.43 (6.29) | <0.001 |
| Age group (%) |  |  |  | <0.001 |
| 80-90 | 5569 (59.91) | 4092 (57.81) | 1477 (66.62) |  |
| 90-100 | 2749 (29.58) | 2180 (30.80) | 569 (25.67) |  |
| 100+ | 977 (10.51) | 806 (11.39) | 171 ( 7.71) | 0.001 |
| Follow-up time (mean (SD)) | 5.45 (4.95) | 5.35 (4.95) | 5.76 (4.94) |  |
| Cognitive impairment (%) |  |  |  | >0.999 |
| no | 3852 (41.44) | 2933 (41.44) | 919 (41.45) |  |
| yes | 5443 (58.56) | 4145 (58.56) | 1298 (58.55) |  |
| education (%) |  |  |  | <0.001 |
| illiteracy | 5760 (61.97) | 4235 (59.83) | 1525 (68.79) |  |
| literacy | 3535 (38.03) | 2843 (40.17) | 692 (31.21) |  |
| Gender (%) |  |  |  | <0.001 |
| female | 4663 (50.17) | 3274 (46.26) | 1389 (62.65) |  |
| male | 4632 (49.83) | 3804 (53.74) | 828 (37.35) |  |
| Marital status (%) |  |  |  | <0.001 |
| no | 106 ( 1.14) | 101 ( 1.43) | 5 ( 0.23) |  |
| yes | 9189 (98.86) | 6977 (98.57) | 2212 (99.77) |  |
| Residence (%) |  |  |  | <0.001 |
| urban | 4266 (45.90) | 3358 (47.44) | 908 (40.96) |  |
| rural | 5029 (54.10) | 3720 (52.56) | 1309 (59.04) |  |
| Smoke (%) |  |  |  | <0.001 |
| no | 5778 (62.16) | 4263 (60.23) | 1515 (68.34) |  |
| current | 2155 (23.18) | 1720 (24.30) | 435 (19.62) |  |
| former | 1362 (14.65) | 1095 (15.47) | 267 (12.04) |  |
| Drink (%) |  |  |  | <0.001 |
| no | 6171 (66.39) | 4615 (65.20) | 1556 (70.18) |  |
| current | 2156 (23.20) | 1672 (23.62) | 484 (21.83) |  |
| former | 968 (10.41) | 791 (11.18) | 177 ( 7.98) |  |
| Exercise (%) |  |  |  | 0.025 |
| no | 5828 (62.70) | 4393 (62.07) | 1435 (64.73) |  |
| yes | 3467 (37.30) | 2685 (37.93) | 782 (35.27) |  |
| ADL disability (%) |  |  |  | <0.001 |
| no | 8026 (86.35) | 5942 (83.95) | 2084 (94.00) |  |
| yes | 1269 (13.65) | 1136 (16.05) | 133 ( 6.00) |  |
| BMI (mean (SD)) | 19.99 (4.24) | 20.10 (4.28) | 19.63 (4.09) | <0.001 |
| Hypertension(%) |  |  |  | 0.685 |
| no | 7805 (83.97) | 5950 (84.06) | 1855 (83.67) |  |
| yes | 1490 (16.03) | 1128 (15.94) | 362 (16.33) |  |
| Diabete (%) |  |  |  | 0.008 |
| no | 9179 (98.75) | 6977 (98.57) | 2202 (99.32) |  |
| yes | 116 ( 1.25) | 101 ( 1.43) | 15 ( 0.68) |  |
| Heart disease (%) |  |  |  | 0.44 |
| no | 8604 (92.57) | 6543 (92.44) | 2061 (92.96) |  |
| yes | 691 ( 7.43) | 535 ( 7.56) | 156 ( 7.04) |  |
| Cerebrovascular disease (%) |  |  |  | <0.001 |
| no | 9004 (96.87) | 6823 (96.40) | 2181 (98.38) |  |
| yes | 291 ( 3.13) | 255 ( 3.60) | 36 ( 1.62) |  |
| Respiratory disease (%) |  |  |  | 0.139 |
| no | 8235 (88.60) | 6251 (88.32) | 1984 (89.49) |  |
| yes | 1060 (11.40) | 827 (11.68) | 233 (10.51) |  |
| Cancer (%) |  |  |  | 0.07 |
| no | 9271 (99.74) | 7064 (99.80) | 2207 (99.55) |  |
| yes | 24 ( 0.26) | 14 ( 0.20) | 10 ( 0.45) |  |
| Comorbidity (%) |  |  |  | 0.118 |
| no | 6397 (68.82) | 4841 (68.40) | 1556 (70.18) |  |
| yes | 2898 (31.18) | 2237 (31.60) | 661 (29.82) |  |
| MMSE scores (mean (SD)) | 27.64 (1.88) | 27.60 (1.90) | 27.78 (1.81) | <0.001 |

**Table S3. Baseline characteristics of the cohort with cut-off value 24 for MMSE scores from main financial support, 1998-2018.**

|  |  | main financial support | |  |
| --- | --- | --- | --- | --- |
|  | Overall | No | Yes | P value |
|  | N=9295 | N=3248 | N=6047 |  |
| Age (mean (SD)) | 88.35 (6.66) | 86.85 (6.19) | 89.16 (6.77) | <0.001 |
| Age group (%) |  |  |  | <0.001 |
| 80-90 | 5569 (59.91) | 2226 (68.53) | 3343 (55.28) |  |
| 90-100 | 2749 (29.58) | 822 (25.31) | 1927 (31.87) |  |
| 100+ | 977 (10.51) | 200 ( 6.16) | 777 (12.85) |  |
| Follow-up time (mean (SD)) | 5.45 (4.95) | 6.48 (5.72) | 4.89 (4.38) | <0.001 |
| Cognitive impairment (%) |  |  |  | <0.001 |
| no | 3852 (41.44) | 1652 (50.86) | 2200 (36.38) |  |
| yes | 5443 (58.56) | 1596 (49.14) | 3847 (63.62) |  |
| education (%) |  |  |  | <0.001 |
| illiteracy | 5760 (61.97) | 1451 (44.67) | 4309 (71.26) |  |
| literacy | 3535 (38.03) | 1797 (55.33) | 1738 (28.74) |  |
| Gender (%) |  |  |  | <0.001 |
| female | 4663 (50.17) | 1078 (33.19) | 3585 (59.29) |  |
| male | 4632 (49.83) | 2170 (66.81) | 2462 (40.71) |  |
| Marital status (%) |  |  |  | <0.001 |
| no | 106 ( 1.14) | 102 ( 3.14) | 4 ( 0.07) |  |
| yes | 9189 (98.86) | 3146 (96.86) | 6043 (99.93) |  |
| Residence (%) |  |  |  | <0.001 |
| urban | 4266 (45.90) | 2152 (66.26) | 2114 (34.96) |  |
| rural | 5029 (54.10) | 1096 (33.74) | 3933 (65.04) |  |
| Smoke (%) |  |  |  | <0.001 |
| no | 5778 (62.16) | 1723 (53.05) | 4055 (67.06) |  |
| current | 2155 (23.18) | 873 (26.88) | 1282 (21.20) |  |
| former | 1362 (14.65) | 652 (20.07) | 710 (11.74) |  |
| Drink (%) |  |  |  | <0.001 |
| no | 6171 (66.39) | 2019 (62.16) | 4152 (68.66) |  |
| current | 2156 (23.20) | 812 (25.00) | 1344 (22.23) |  |
| former | 968 (10.41) | 417 (12.84) | 551 ( 9.11) |  |
| Exercise (%) |  |  |  | <0.001 |
| no | 5828 (62.70) | 1615 (49.72) | 4213 (69.67) |  |
| yes | 3467 (37.30) | 1633 (50.28) | 1834 (30.33) |  |
| ADL disability (%) |  |  |  | 0.903 |
| no | 8026 (86.35) | 2807 (86.42) | 5219 (86.31) |  |
| yes | 1269 (13.65) | 441 (13.58) | 828 (13.69) |  |
| BMI (mean (SD)) | 19.99 (4.24) | 20.72 (4.36) | 19.59 (4.12) | <0.001 |
| Hypertension (%) |  |  |  | <0.001 |
| no | 7805 (83.97) | 2668 (82.14) | 5137 (84.95) |  |
| yes | 1490 (16.03) | 580 (17.86) | 910 (15.05) |  |
| Diabetes (%) |  |  |  | <0.001 |
| no | 9179 (98.75) | 3179 (97.88) | 6000 (99.22) |  |
| yes | 116 ( 1.25) | 69 ( 2.12) | 47 ( 0.78) |  |
| Heart disease (%) |  |  |  | <0.001 |
| no | 8604 (92.57) | 2868 (88.30) | 5736 (94.86) |  |
| yes | 691 ( 7.43) | 380 (11.70) | 311 ( 5.14) |  |
| Cerebrovascular disease (%) |  |  |  | <0.001 |
| no | 9004 (96.87) | 3104 (95.57) | 5900 (97.57) |  |
| yes | 291 ( 3.13) | 144 ( 4.43) | 147 ( 2.43) |  |
| Respiratory disease (%) |  |  |  | <0.001 |
| no | 8235 (88.60) | 2797 (86.11) | 5438 (89.93) |  |
| yes | 1060 (11.40) | 451 (13.89) | 609 (10.07) |  |
| Cancer (%) |  |  |  | 0.009 |
| no | 9271 (99.74) | 3233 (99.54) | 6038 (99.85) |  |
| yes | 24 ( 0.26) | 15 ( 0.46) | 9 ( 0.15) |  |
| Comorbidity (%) |  |  |  | <0.001 |
| no | 6397 (68.82) | 2036 (62.68) | 4361 (72.12) |  |
| yes | 2898 (31.18) | 1212 (37.32) | 1686 (27.88) |  |
| MMSE scores (mean (SD)) | 27.64 (1.88) | 27.94 (1.82) | 27.49 (1.89) | <0.001 |

**Table S4. Baseline characteristics of the cohort with cut-off value 24 for MMSE scores from living or being visited, 1998-2018.**

|  |  | living or being visited | |  |
| --- | --- | --- | --- | --- |
|  | Overall | No | Yes | p |
|  | N=9295 | N=704 | N=8591 |  |
| Age (mean (SD)) | 88.35 (6.66) | 87.68 (6.72) | 88.41 (6.66) | 0.005 |
| Age group (%) |  |  |  |  |
| 80-90 | 5569 (59.91) | 467 (66.34) | 5102 (59.39) | 0.001 |
| 90-100 | 2749 (29.58) | 173 (24.57) | 2576 (29.98) |  |
| 100+ | 977 (10.51) | 64 ( 9.09) | 913 (10.63) |  |
| Follow-up time (mean (SD)) | 5.45 (4.95) | 5.67 (5.55) | 5.43 (4.90) | 0.212 |
| Cognitive impairment (%) |  |  |  | 0.31 |
| no | 3852 (41.44) | 305 (43.32) | 3547 (41.29) |  |
| yes | 5443 (58.56) | 399 (56.68) | 5044 (58.71) |  |
| education (%) |  |  |  | 0.951 |
| illiteracy | 5760 (61.97) | 435 (61.79) | 5325 (61.98) |  |
| literacy | 3535 (38.03) | 269 (38.21) | 3266 (38.02) |  |
| Gender (%) |  |  |  | 0.105 |
| female | 4663 (50.17) | 332 (47.16) | 4331 (50.41) |  |
| male | 4632 (49.83) | 372 (52.84) | 4260 (49.59) |  |
| Marital status (%) |  |  |  | <0.001 |
| no | 106 ( 1.14) | 96 (13.64) | 10 ( 0.12) |  |
| yes | 9189 (98.86) | 608 (86.36) | 8581 (99.88) |  |
| Residence (%) |  |  |  | <0.001 |
| urban | 4266 (45.90) | 413 (58.66) | 3853 (44.85) |  |
| rural | 5029 (54.10) | 291 (41.34) | 4738 (55.15) |  |
| Smoke (%) |  |  |  | 0.011 |
| no | 5778 (62.16) | 403 (57.24) | 5375 (62.57) |  |
| current | 2155 (23.18) | 193 (27.41) | 1962 (22.84) |  |
| former | 1362 (14.65) | 108 (15.34) | 1254 (14.60) |  |
| Drink (%) |  |  |  | 0.396 |
| no | 6171 (66.39) | 451 (64.06) | 5720 (66.58) |  |
| current | 2156 (23.20) | 175 (24.86) | 1981 (23.06) |  |
| former | 968 (10.41) | 78 (11.08) | 890 (10.36) |  |
| Exercise (%) |  |  |  | 0.47 |
| no | 5828 (62.70) | 432 (61.36) | 5396 (62.81) |  |
| yes | 3467 (37.30) | 272 (38.64) | 3195 (37.19) |  |
| ADL disability (%) |  |  |  | 0.226 |
| no | 8026 (86.35) | 619 (87.93) | 7407 (86.22) |  |
| yes | 1269 (13.65) | 85 (12.07) | 1184 (13.78) |  |
| BMI (mean (SD)) | 19.99 (4.24) | 19.79 (4.17) | 20.00 (4.24) | 0.202 |
| Hypertension (%) |  |  |  | 0.567 |
| no | 7805 (83.97) | 597 (84.80) | 7208 (83.90) |  |
| yes | 1490 (16.03) | 107 (15.20) | 1383 (16.10) |  |
| Diabetes (%) |  |  |  | 0.42 |
| no | 9179 (98.75) | 698 (99.15) | 8481 (98.72) |  |
| yes | 116 ( 1.25) | 6 ( 0.85) | 110 ( 1.28) |  |
| Heart disease (%) |  |  |  | 0.129 |
| no | 8604 (92.57) | 641 (91.05) | 7963 (92.69) |  |
| yes | 691 ( 7.43) | 63 ( 8.95) | 628 ( 7.31) |  |
| Cerebrovascular disease (%) |  |  |  | 0.425 |
| no | 9004 (96.87) | 686 (97.44) | 8318 (96.82) |  |
| yes | 291 ( 3.13) | 18 ( 2.56) | 273 ( 3.18) |  |
| Respiratory disease (%) |  |  |  | 0.034 |
| no | 8235 (88.60) | 606 (86.08) | 7629 (88.80) |  |
| yes | 1060 (11.40) | 98 (13.92) | 962 (11.20) |  |
| Cancer (%) |  |  |  | 0.806 |
| no | 9271 (99.74) | 703 (99.86) | 8568 (99.73) |  |
| yes | 24 ( 0.26) | 1 ( 0.14) | 23 ( 0.27) |  |
| Comorbidity (%) |  |  |  | 0.799 |
| no | 6397 (68.82) | 481 (68.32) | 5916 (68.86) |  |
| yes | 2898 (31.18) | 223 (31.68) | 2675 (31.14) |  |
| MMSE scores (mean (SD)) | 27.64 (1.88) | 27.60 (1.87) | 27.65 (1.88) | 0.533 |

**Table S5. Baseline characteristics of the cohort with cut-off value 18 for MMSE scores from housework or childcare, 1998-2018.**

|  |  | housework or childcare | |  |
| --- | --- | --- | --- | --- |
|  | Overall | No | Yes | P value |
|  | N=11976 | N=9287 | N=2689 |  |
| Age (mean (SD)) | 89.08 (6.91) | 89.42 (6.99) | 87.92 (6.52) | <0.001 |
| Age group (%) |  |  |  | <0.001 |
| 80-90 | 6659 (55.60) | 4943 (53.22) | 1716 (63.82) |  |
| 90-100 | 3719 (31.05) | 3004 (32.35) | 715 (26.59) |  |
| 100+ | 1598 (13.34) | 1340 (14.43) | 258 ( 9.59) |  |
| Follow-up time (mean (SD)) | 6.15 (5.34) | 6.00 (5.33) | 6.67 (5.31) | <0.001 |
| Cognitive impairment (%) |  |  |  | 0.136 |
| no | 7198 (60.10) | 5548 (59.74) | 1650 (61.36) |  |
| yes | 4778 (39.90) | 3739 (40.26) | 1039 (38.64) |  |
| education (%) |  |  |  | <0.001 |
| illiteracy | 7992 (66.73) | 6046 (65.10) | 1946 (72.37) |  |
| literacy | 3984 (33.27) | 3241 (34.90) | 743 (27.63) |  |
| Gender (%) |  |  |  | <0.001 |
| female | 6554 (54.73) | 4778 (51.45) | 1776 (66.05) |  |
| male | 5422 (45.27) | 4509 (48.55) | 913 (33.95) |  |
| Marital status (%) |  |  |  | <0.001 |
| no | 137 ( 1.14) | 132 ( 1.42) | 5 ( 0.19) |  |
| yes | 11839 (98.86) | 9155 (98.58) | 2684 (99.81) |  |
| Residence (%) |  |  |  | <0.001 |
| urban | 5285 (44.13) | 4235 (45.60) | 1050 (39.05) |  |
| rural | 6691 (55.87) | 5052 (54.40) | 1639 (60.95) |  |
| Smoke (%) |  |  |  | <0.001 |
| no | 7722 (64.48) | 5834 (62.82) | 1888 (70.21) |  |
| current | 2632 (21.98) | 2132 (22.96) | 500 (18.59) |  |
| former | 1622 (13.54) | 1321 (14.22) | 301 (11.19) |  |
| Drink (%) |  |  |  | <0.001 |
| no | 8067 (67.36) | 6167 (66.40) | 1900 (70.66) |  |
| current | 2694 (22.49) | 2118 (22.81) | 576 (21.42) |  |
| former | 1215 (10.15) | 1002 (10.79) | 213 ( 7.92) |  |
| Exercise (%) |  |  |  | 0.249 |
| no | 7854 (65.58) | 6065 (65.31) | 1789 (66.53) |  |
| yes | 4122 (34.42) | 3222 (34.69) | 900 (33.47) |  |
| ADL disability (%) |  |  |  | <0.001 |
| no | 10004 (83.53) | 7486 (80.61) | 2518 (93.64) |  |
| yes | 1972 (16.47) | 1801 (19.39) | 171 ( 6.36) | <0.001 |
| BMI (mean (SD)) | 19.76 (4.21) | 19.84 (4.25) | 19.48 (4.07) |  |
| Hypertension (%) |  |  |  | 0.763 |
| no | 10054 (83.95) | 7791 (83.89) | 2263 (84.16) |  |
| yes | 1922 (16.05) | 1496 (16.11) | 426 (15.84) |  |
| Diabetes (%) |  |  |  | 0.005 |
| no | 11836 (98.83) | 9164 (98.68) | 2672 (99.37) |  |
| yes | 140 ( 1.17) | 123 ( 1.32) | 17 ( 0.63) |  |
| Heart disease (%) |  |  |  | 0.168 |
| no | 11113 (92.79) | 8601 (92.61) | 2512 (93.42) |  |
| yes | 863 ( 7.21) | 686 ( 7.39) | 177 ( 6.58) |  |
| Cerebrovascular disease (%) |  |  |  | <0.001 |
| no | 11599 (96.85) | 8959 (96.47) | 2640 (98.18) |  |
| yes | 377 ( 3.15) | 328 ( 3.53) | 49 ( 1.82) |  |
| Respiratory disease (%) |  |  |  | 0.031 |
| no | 10647 (88.90) | 8225 (88.56) | 2422 (90.07) |  |
| yes | 1329 (11.10) | 1062 (11.44) | 267 ( 9.93) |  |
| Cancer (%) |  |  |  | 0.183 |
| no | 11947 (99.76) | 9268 (99.80) | 2679 (99.63) |  |
| yes | 29 ( 0.24) | 19 ( 0.20) | 10 ( 0.37) |  |
| Comorbidity (%) |  |  |  | 0.012 |
| no | 8272 (69.07) | 6361 (68.49) | 1911 (71.07) |  |
| yes | 3704 (30.93) | 2926 (31.51) | 778 (28.93) |  |
| MMSE scores (mean (SD)) | 26.13 (3.36) | 26.00 (3.42) | 26.60 (3.10) | <0.001 |

**Table S6. Baseline characteristics of the cohort with cut-off value 18 for MMSE scores from main financial support, 1998-2018.**

|  |  | main financial support | |  |
| --- | --- | --- | --- | --- |
|  | Overall | No | Yes | P value |
|  | N=11976 | N=3804 | N=8172 |  |
| Age (mean (SD)) | 89.08 (6.91) | 87.32 (6.45) | 89.91 (6.97) | <0.001 |
| Age group (%) |  |  |  |  |
| 80-90 | 6659 (55.60) | 2509 (65.96) | 4150 (50.78) | <0.001 |
| 90-100 | 3719 (31.05) | 1001 (26.31) | 2718 (33.26) |  |
| 100+ | 1598 (13.34) | 294 ( 7.73) | 1304 (15.96) |  |
| Follow-up time (mean (SD)) | 6.15 (5.34) | 7.22 (6.02) | 5.65 (4.91) | <0.001 |
| Cognitive impairment (%) |  |  |  |  |
| no | 7198 (60.10) | 2603 (68.43) | 4595 (56.23) | <0.001 |
| yes | 4778 (39.90) | 1201 (31.57) | 3577 (43.77) |  |
| education (%) |  |  |  |  |
| illiteracy | 7992 (66.73) | 1858 (48.84) | 6134 (75.06) | <0.001 |
| literacy | 3984 (33.27) | 1946 (51.16) | 2038 (24.94) |  |
| Gender (%) |  |  |  |  |
| female | 6554 (54.73) | 1395 (36.67) | 5159 (63.13) | <0.001 |
| male | 5422 (45.27) | 2409 (63.33) | 3013 (36.87) |  |
| Marital status (%) |  |  |  |  |
| no | 137 ( 1.14) | 132 ( 3.47) | 5 ( 0.06) | <0.001 |
| yes | 11839 (98.86) | 3672 (96.53) | 8167 (99.94) |  |
| Residence (%) |  |  |  |  |
| urban | 5285 (44.13) | 2490 (65.46) | 2795 (34.20) | <0.001 |
| rural | 6691 (55.87) | 1314 (34.54) | 5377 (65.80) |  |
| Smoke (%) |  |  |  |  |
| no | 7722 (64.48) | 2092 (54.99) | 5630 (68.89) | <0.001 |
| current | 2632 (21.98) | 999 (26.26) | 1633 (19.98) |  |
| former | 1622 (13.54) | 713 (18.74) | 909 (11.12) |  |
| Drink (%) |  |  |  |  |
| no | 8067 (67.36) | 2403 (63.17) | 5664 (69.31) | <0.001 |
| current | 2694 (22.49) | 926 (24.34) | 1768 (21.63) |  |
| former | 1215 (10.15) | 475 (12.49) | 740 ( 9.06) |  |
| Exercise (%) |  |  |  |  |
| no | 7854 (65.58) | 1970 (51.79) | 5884 (72.00) | <0.001 |
| yes | 4122 (34.42) | 1834 (48.21) | 2288 (28.00) |  |
| ADL disability (%) |  |  |  |  |
| no | 10004 (83.53) | 3223 (84.73) | 6781 (82.98) | 0.018 |
| yes | 1972 (16.47) | 581 (15.27) | 1391 (17.02) |  |
| BMI (mean (SD)) | 19.76 (4.21) | 20.56 (4.35) | 19.39 (4.09) | <0.001 |
| Hypertension (%) |  |  |  | <0.001 |
| no | 10054 (83.95) | 3127 (82.20) | 6927 (84.77) |  |
| yes | 1922 (16.05) | 677 (17.80) | 1245 (15.23) |  |
| Diabetes (%) |  |  |  | <0.001 |
| no | 11836 (98.83) | 3724 (97.90) | 8112 (99.27) |  |
| yes | 140 ( 1.17) | 80 ( 2.10) | 60 ( 0.73) |  |
| Heart disease (%) |  |  |  | <0.001 |
| no | 11113 (92.79) | 3363 (88.41) | 7750 (94.84) |  |
| yes | 863 ( 7.21) | 441 (11.59) | 422 ( 5.16) |  |
| Cerebrovascular disease (%) |  |  |  | <0.001 |
| no | 11599 (96.85) | 3637 (95.61) | 7962 (97.43) |  |
| yes | 377 ( 3.15) | 167 ( 4.39) | 210 ( 2.57) |  |
| Respiratory disease (%) |  |  |  | <0.001 |
| no | 10647 (88.90) | 3281 (86.25) | 7366 (90.14) |  |
| yes | 1329 (11.10) | 523 (13.75) | 806 ( 9.86) |  |
| Cancer (%) |  |  |  | 0.004 |
| no | 11947 (99.76) | 3787 (99.55) | 8160 (99.85) |  |
| yes | 29 ( 0.24) | 17 ( 0.45) | 12 ( 0.15) |  |
| Comorbidity (%) |  |  |  | <0.001 |
| no | 8272 (69.07) | 2390 (62.83) | 5882 (71.98) |  |
| yes | 3704 (30.93) | 1414 (37.17) | 2290 (28.02) |  |
| MMSE scores (mean (SD)) | 26.13 (3.36) | 26.93 (3.03) | 25.76 (3.44) | <0.001 |

**Table S7. Baseline characteristics of the cohort with cut-off value 18 for MMSE scores from living or being visited, 1998-2018.**

|  |  | living or being visited | |  |
| --- | --- | --- | --- | --- |
|  | Overall | No | Yes | P value |
|  | N=11976 | N=920 | N=11056 |  |
| Age (mean (SD)) | 89.08 (6.91) | 88.47 (7.13) | 89.14 (6.89) | 0.005 |
| Age group (%) |  |  |  | 0.001 |
| 80-90 | 6659 (55.60) | 566 (61.52) | 6093 (55.11) |  |
| 90-100 | 3719 (31.05) | 242 (26.30) | 3477 (31.45) |  |
| 100+ | 1598 (13.34) | 112 (12.17) | 1486 (13.44) | 0.005 |
| Follow-up time (mean (SD)) | 6.15 (5.34) | 6.63 (6.13) | 6.11 (5.26) |  |
| Cognitive impairment (%) |  |  |  | 0.219 |
| no | 7198 (60.10) | 571 (62.07) | 6627 (59.94) |  |
| yes | 4778 (39.90) | 349 (37.93) | 4429 (40.06) |  |
| education (%) |  |  |  | 0.802 |
| illiteracy | 7992 (66.73) | 610 (66.30) | 7382 (66.77) |  |
| literacy | 3984 (33.27) | 310 (33.70) | 3674 (33.23) |  |
| Gender (%) |  |  |  | 0.113 |
| female | 6554 (54.73) | 480 (52.17) | 6074 (54.94) |  |
| male | 5422 (45.27) | 440 (47.83) | 4982 (45.06) |  |
| Marital status (%) |  |  |  | <0.001 |
| no | 137 ( 1.14) | 124 (13.48) | 13 ( 0.12) |  |
| yes | 11839 (98.86) | 796 (86.52) | 11043 (99.88) |  |
| Residence (%) |  |  |  | <0.001 |
| urban | 5285 (44.13) | 518 (56.30) | 4767 (43.12) |  |
| rural | 6691 (55.87) | 402 (43.70) | 6289 (56.88) |  |
| Smoke (%) |  |  |  | 0.002 |
| no | 7722 (64.48) | 557 (60.54) | 7165 (64.81) |  |
| current | 2632 (21.98) | 244 (26.52) | 2388 (21.60) |  |
| former | 1622 (13.54) | 119 (12.93) | 1503 (13.59) |  |
| Drink (%) |  |  |  | 0.516 |
| no | 8067 (67.36) | 604 (65.65) | 7463 (67.50) |  |
| current | 2694 (22.49) | 218 (23.70) | 2476 (22.40) |  |
| former | 1215 (10.15) | 98 (10.65) | 1117 (10.10) |  |
| Exercise (%) |  |  |  | 0.621 |
| no | 7854 (65.58) | 596 (64.78) | 7258 (65.65) |  |
| yes | 4122 (34.42) | 324 (35.22) | 3798 (34.35) |  |
| ADL disability (%) |  |  |  | 0.042 |
| no | 10004 (83.53) | 791 (85.98) | 9213 (83.33) |  |
| yes | 1972 (16.47) | 129 (14.02) | 1843 (16.67) |  |
| BMI (mean (SD)) | 19.76 (4.21) | 19.58 (4.17) | 19.77 (4.22) | 0.172 |
| Hypertension (%) |  |  |  |  |
| no | 10054 (83.95) | 776 (84.35) | 9278 (83.92) | 0.768 |
| yes | 1922 (16.05) | 144 (15.65) | 1778 (16.08) |  |
| Diabetes (%) |  |  |  |  |
| no | 11836 (98.83) | 911 (99.02) | 10925 (98.82) | 0.689 |
| yes | 140 ( 1.17) | 9 ( 0.98) | 131 ( 1.18) |  |
| Heart disease (%) |  |  |  |  |
| no | 11113 (92.79) | 837 (90.98) | 10276 (92.95) | 0.032 |
| yes | 863 ( 7.21) | 83 ( 9.02) | 780 ( 7.05) |  |
| Cerebrovascular disease (%) |  |  |  |  |
| no | 11599 (96.85) | 900 (97.83) | 10699 (96.77) | 0.096 |
| yes | 377 ( 3.15) | 20 ( 2.17) | 357 ( 3.23) |  |
| Respiratory disease (%) |  |  |  |  |
| no | 10647 (88.90) | 788 (85.65) | 9859 (89.17) | 0.001 |
| yes | 1329 (11.10) | 132 (14.35) | 1197 (10.83) |  |
| Cancer (%) |  |  |  |  |
| no | 11947 (99.76) | 917 (99.67) | 11030 (99.76) | 0.849 |
| yes | 29 ( 0.24) | 3 ( 0.33) | 26 ( 0.24) |  |
| Comorbidity (%) |  |  |  |  |
| no | 8272 (69.07) | 614 (66.74) | 7658 (69.27) | 0.12 |
| yes | 3704 (30.93) | 306 (33.26) | 3398 (30.73) |  |
| MMSE scores (mean (SD)) | 26.13 (3.36) | 26.05 (3.35) | 26.14 (3.36) | 0.443 |

**Table S8. Association between intergenerational relationships and cognitive impairment with cut-off value 18 for MMSE scores.**

|  | Model 1 |  | Model 2 |  | Model 3 |  |
| --- | --- | --- | --- | --- | --- | --- |
|  | Crude HR (95% CI) | P value | HR (95% CI) | P value | HR (95% CI) | P value |
| main financial support |  | <0.001 |  | <0.001 |  | <0.001 |
| No | 1 Reference |  | 1 Reference |  | 1 Reference |  |
| Yes | 3.42 (3.13, 3.75) |  | 2.86 (2.60, 3.14) |  | 2.75 (2.50, 3.02) |  |
| living or being visited |  | 0.168 |  | 0.807 |  | 0.648 |
| No | 1 Reference |  | 1 Reference |  | 1 Reference |  |
| Yes | 1.08 (0.97, 1.20) |  | 1.01 (0.91, 1.14) |  | 1.03 (0.92, 1.15) |  |
| Housework or childcare |  | <0.001 |  | <0.001 |  | <0.001 |
| No | 1 Reference |  | 1 Reference |  | 1 Reference |  |
| Yes | 0.79 (0.74, 0.85) |  | 0.79 (0.74, 0.85) |  | 0.85 (0.79, 0.92) |  |

Abbreviations: HR=hazard ratio; CI=confidence interval.

Model 2 were adjusted for age, gender, residence, marital status and education.

Model 3 were further adjusted for smoking, drinking status, exercise, ADL disability, BMI, comorbidity and baseline Chinese MMSE scores.

**Table S9. Association between intergenerational relationships and cognitive impairment with cut-off value 18 for MMSE scores using traditional Cox regression.**

|  | Model1 |  | Model2 |  | Model3 |  |
| --- | --- | --- | --- | --- | --- | --- |
|  | Crude HR (95% CI) | P value | HR (95% CI) | P value | HR (95% CI) | P value |
| main financial support |  | <0.001 |  | <0.001 |  | <0.001 |
| No | 1 Reference |  | 1 Reference |  | 1 Reference |  |
| Yes | 1.67 (1.56, 1.78) |  | 1.23 (1.14, 1.32) |  | 1.16 (1.08, 1.25) |  |
| living or being visited |  | 0.309 |  | 0.636 |  | 0.793 |
| No | 1 Reference |  | 1 Reference |  | 1 Reference |  |
| Yes | 1.06 (0.95, 1.18) |  | 0.97 (0.87, 1.09) |  | 0.98 (0.87, 1.10) | 0.719 |
| Housework or childcare |  | <0.001 |  | <0.001 |  | 0.068 |
| No | 1 Reference |  | 1 Reference |  | 1 Reference |  |
| Yes | 0.84 (0.79, 0.90) |  | 0.86 (0.80, 0.92) |  | 0.94 (0.87, 1.00) |  |

Abbreviations: HR=hazard ratio; CI=confidence interval.

Model 2 were adjusted for age, gender, residence, marital status and education.

Model 3 were further adjusted for smoking, drinking status, exercise, ADL disability, BMI, comorbidity and baseline Chinese MMSE scores.

| 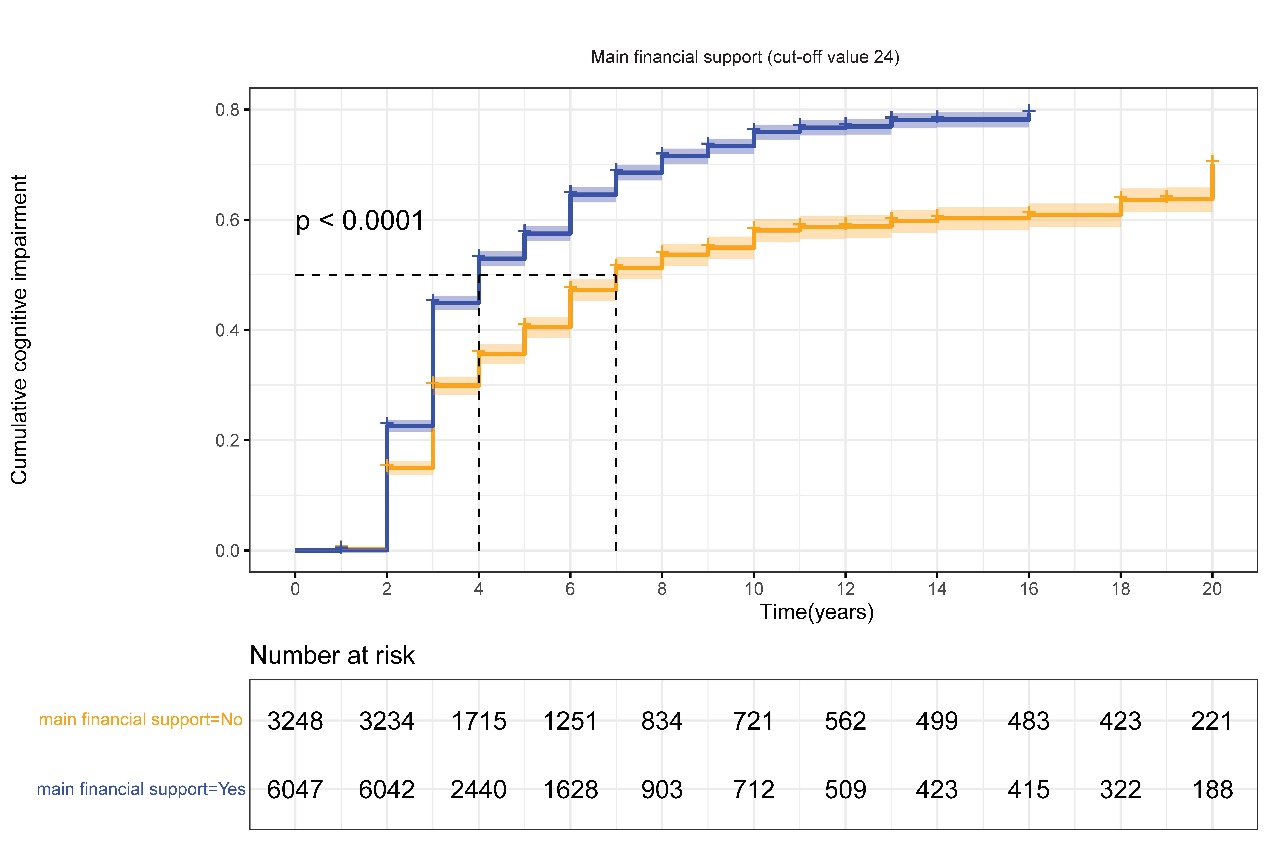 |
| --- |
| 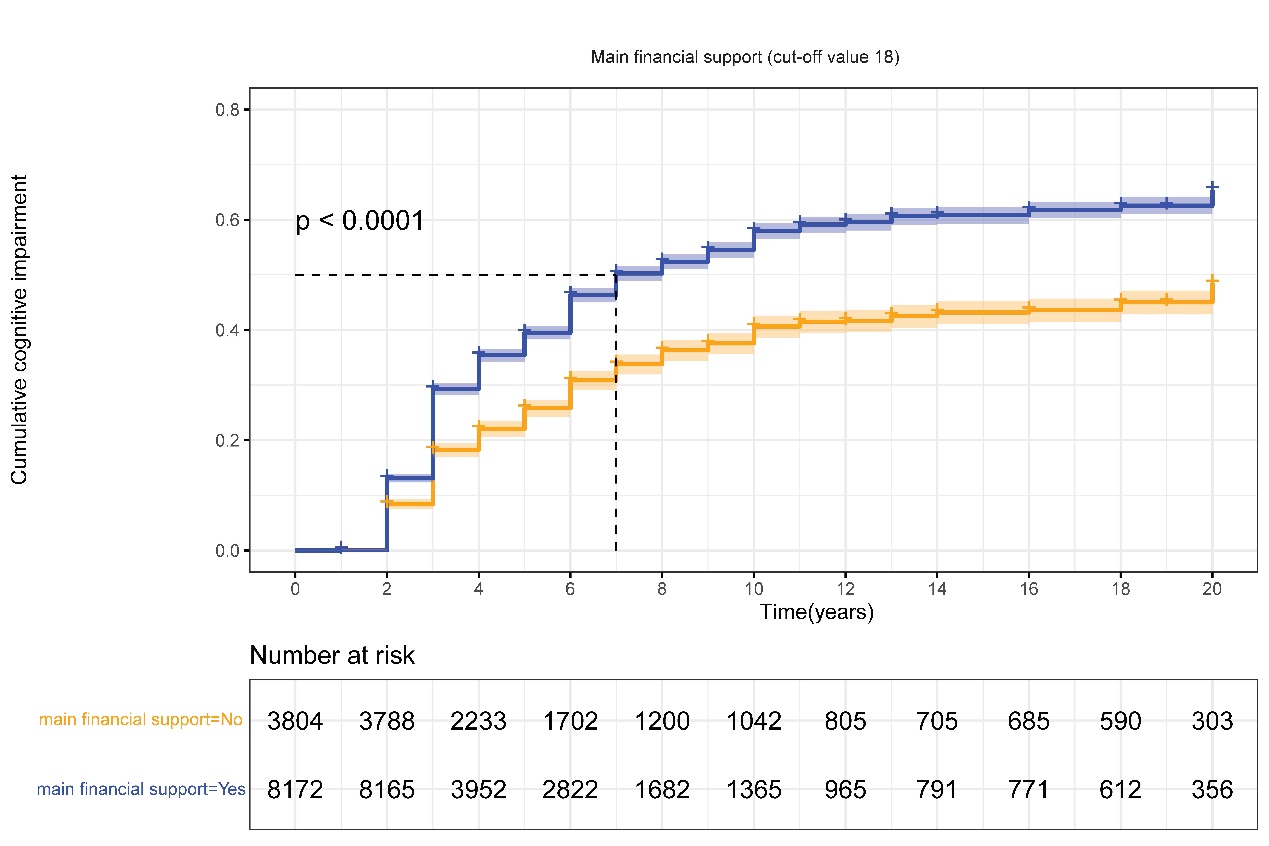 |
| 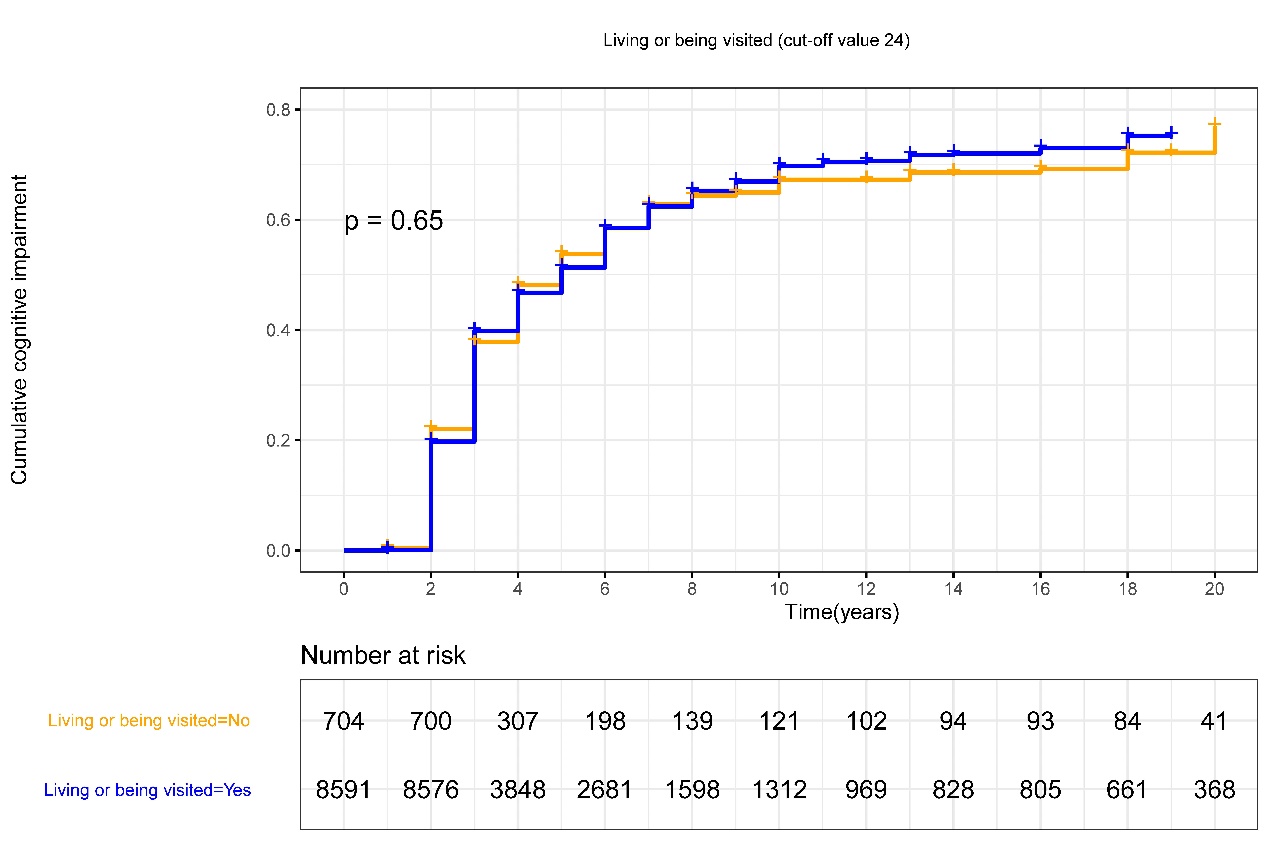 |
| 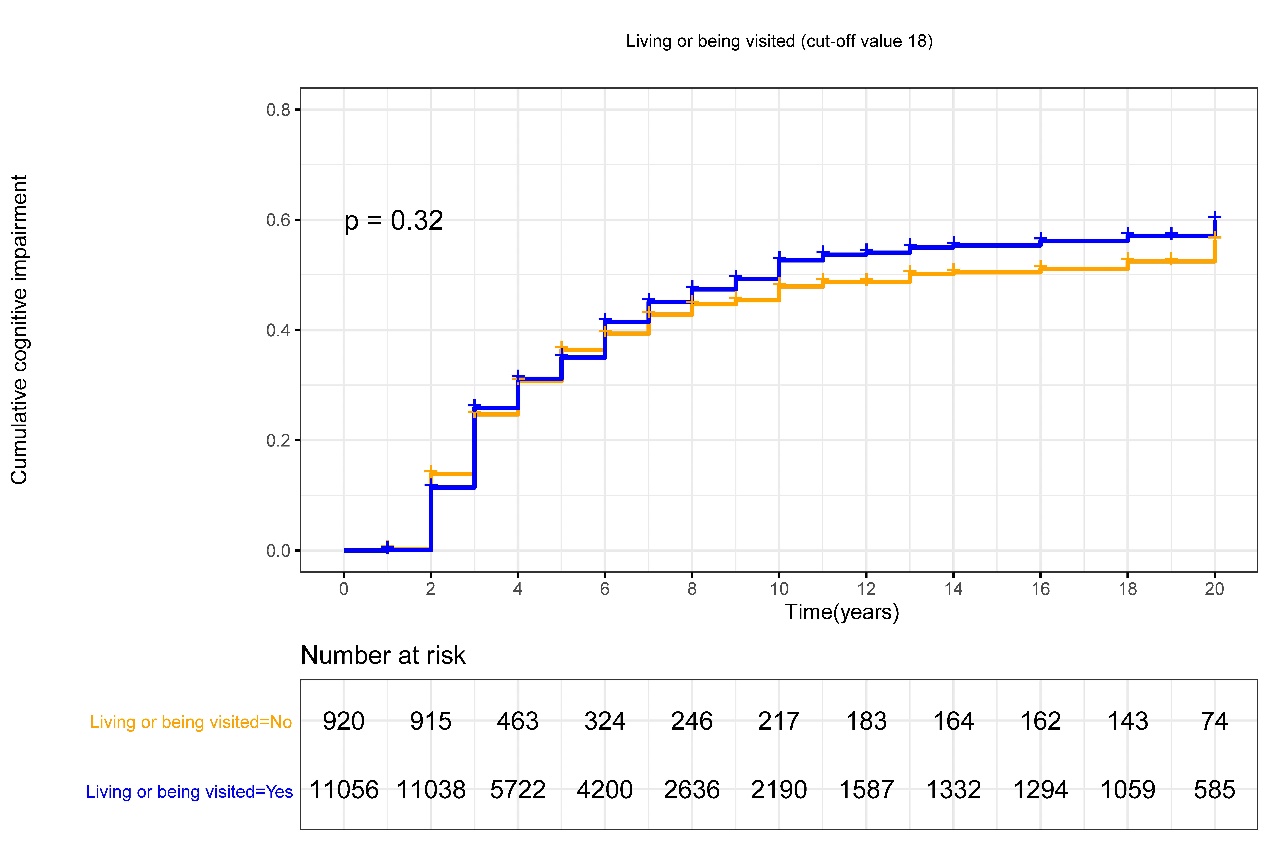 |
| 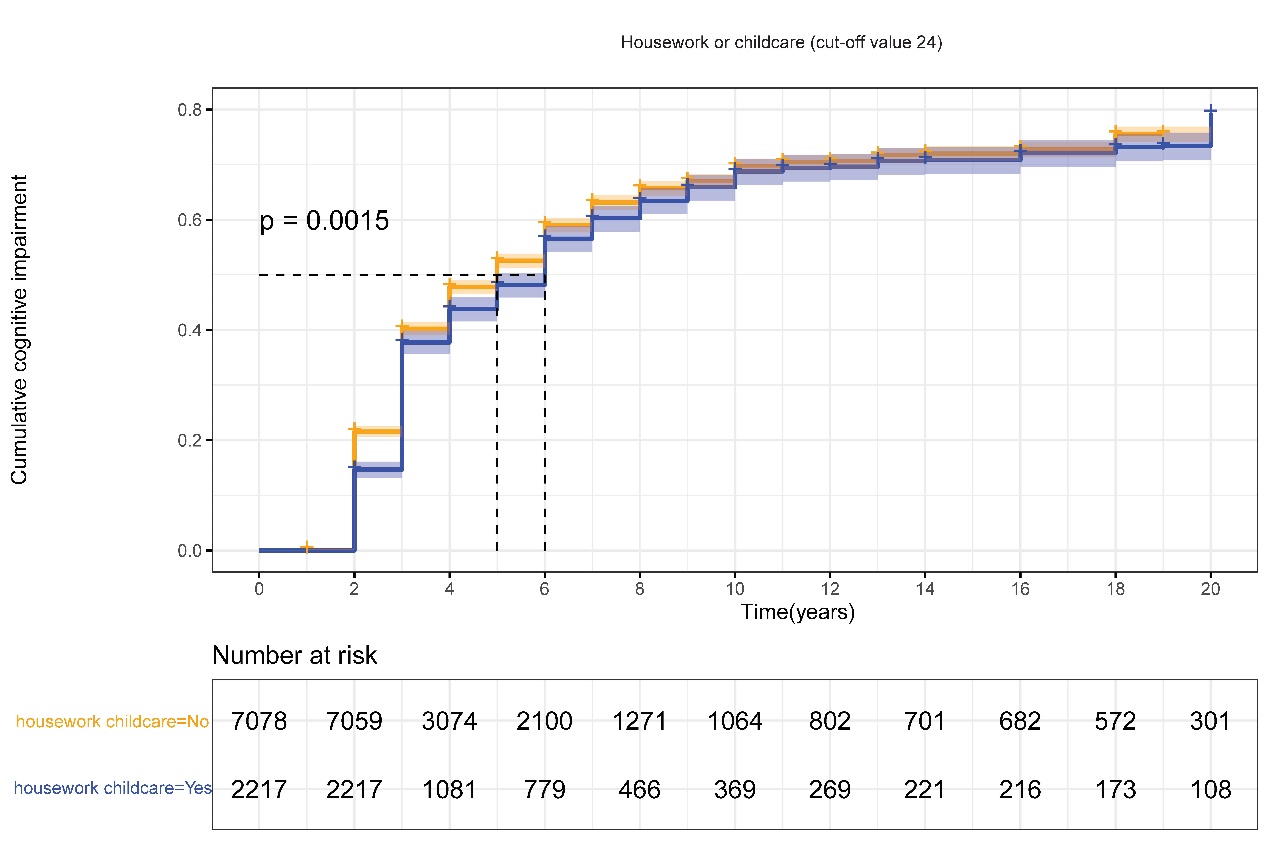 |
| 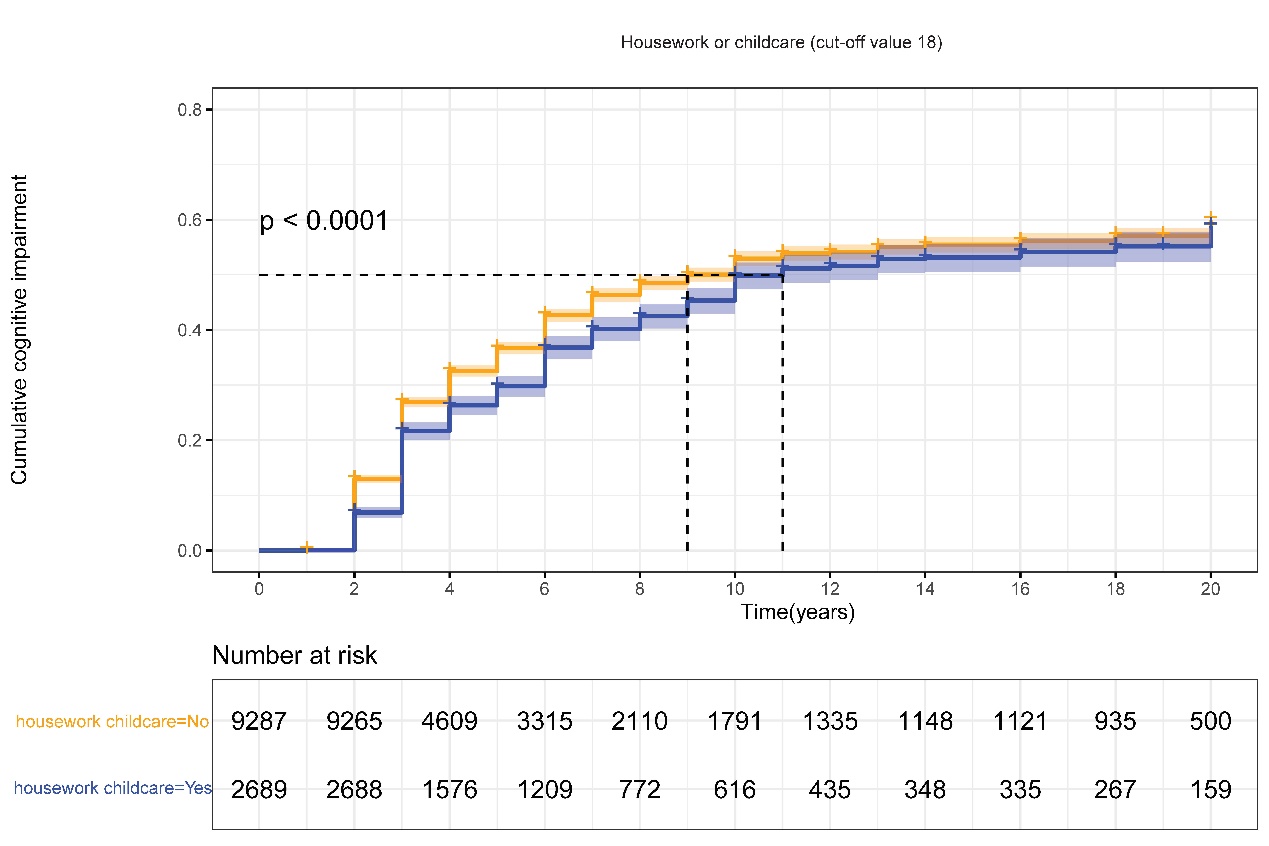 |
| **Figure S1. Cumulative cognitive impairment curves in intergenerational relationships variables.** |
